# The Measurement of Averages and Extremes of Environmental Variables

**DOI:** 10.6028/jres.099.055

**Published:** 1994

**Authors:** C. W. Anderson, K. F. Turkman

**Affiliations:** University of Sheffield, Sheffield, UK; University of Lisbon, Lisbon, Portugal

**Keywords:** averages, extremes, extremal index, Poisson random measures

## Abstract

The relationship between the statistics of environmental measurements averaged over different time scales is related to extreme levels of the variables. Results on the asymptotic joint distributions of extreme averages over different time periods are treated.

## 1. Introduction

Measurements of many environmental variables such as wind speeds, rainfall and the concentrations of atmospheric and aquatic pollutants are generally duration-specific: the actual quantities measured are averages over a specific time interval rather than instantaneous values. Thus concentrations of ozone are typically measured as parts per 100 million averaged over an hour, and wind speeds are routinely recorded as hourly or daily mean speeds. In practice however, the scientists wishing to understand the environmental processes which lie behind such measurements, and the regulatory body which monitors pollution, often wish to deal with characteristics measured over some other time interval: peak concentrations over a day, for example, or high wind gust values, which in practice correspond to peak 3–5 s averages. There is therefore a need to understand the relationship between the statistics of environmental measurements averaged over different time scales, and to relate these statistics to extreme levels of the variables. In this paper, results on the asymptotic joint distributions of extreme averages over different time periods will be treated. These results will make it possible, for example, to link long historical data series containing information about extremes of daily rainfall (sometimes extending back to the early years of the century) to the shorter series of extreme hourly rainfall which have been recorded only in the past 20 or 30 years. Thus important historical information could properly be taken account of in the estimation of floods, something recognised as higly desirable by hydrologists. Another area of application of the results is in the study of the dispersal of airborne pollutants. Here, it is known (see, for example Fakrell and Robins [9]) that instruments used to measure the concentration of pollutants dispersing in a turbulent flow cannot resolve the finest scales present in such flows. Measurements of concentration are therefore invariably obtained only as averages of the characteristics of primary physical interest, and so a statistical theory which links extremes of averages over different ranges would be of great value to scientists working in the area (Mole [10]). One related area is the study of joint distributions of averages and maxima of random sequences. Interest in such distributions is motivated by analysis of extreme winds. For purposes of building design or public safety, it is often important to estimate the speed of the most extreme wind likely to occur at a particular location over a period of years and to do this it is natural to apply the methods of extreme value theory to data on maximum gusts. The precision of estimates obtained may be low due to the limited amount of relevant data—often no more than 10 or 20 years, so it is desirable to try to improve the precision by introducing into the estimation procedure other information relevant to extreme winds. It is natural, thefore, to ask whether the data on gusts could be augmented by that on hourly means, as gusts and means are evidently related. One source of guidance here may be provided by the limit properties of the joint distribution of means and the maxima.

In Sec. 2, we give a summary of the results that exist on the joint limiting forms of sums and maxima of stationary sequences and in Sec. 3 we give some results on the asymptotic joint distributions of extreme averages over different time-periods of sequences which have moving average representations. Possible solutions for the general stationary case are also indicated.

## 2. Extremes and Averages

Let {*X_i_*} be a stationary sequence of random variables with marginal distribution function P(*X_i_* ≤ *x*) = *F*(*x*) and let

$$S_{n}=\sum_{i=1}^{n}X_{i},M_{n}=\underset{1\leq i\leq n}{\max}X_{n},n=1,2,\ldots$$

We study here the joint limiting distributions of

$$\left({\bar{S}}_{n},{\bar{M}}_{n}\right)=\left(\frac{S_{n}-nb_{n}}{a_{n}},\frac{M_{n}-d_{n}}{c_{n}}\right)$$ (1)

as *n* → ∞ for suitable constants *a_n_* > 0, *c_n_* > 0, *b_n_* and *d_n_*.

### Case 1: Light Tailed Case

Assume that *Var* (*X_i_*) < ∞ and *F* ∈ *D* (Λ) or *F* ∈ *D* (Φ_α_), α>2 or *F* ∈ *D* (Ψ_α_), α>0. where Λ(.), Φ_α_(.), Ψ_α_(.) are respectively the Gumbel, Frechet and Weibull distributions.

Then for the associated iid sequence (Chow and Teugels [5])

$$\left({\bar{S}}_{n},{\bar{M}}_{n}\right)\to^{d}\left(N,\Lambda \right),$$

or

$$\begin{array}{l} \left({\bar{S}}_{n},{\bar{M}}_{n}\right)\to^{d}\left(N,\Phi_{\alpha }\right),\alpha >2,\\ \left({\bar{S}}_{n},{\bar{M}}_{n}\right)\to^{d}\left(N,\Psi_{\alpha }\right),\alpha >0, \end{array}$$

where the limit components are independent.

Can dependence amongst *X_i_* modify this limiting independence? As the following theorem (Anderson and Turkman [1]) shows, under quite weak conditions, dependence does not affect the limiting distribution.

***Theorem 2.1***

Assume that {*X_i_*} is strong mixing and has positive extremal index and for some *a_n_*, *c_n_* and *d_n_*,

$${\bar{S}}_{n}=\frac{S_{n}}{a_{n}}\to^{d}N\left(0,1\right),$$

$${\bar{M}}_{n}=\frac{\left(M_{n}-d_{n}\right)}{c_{n}}\to^{d}G,$$

where *G* =Λ or *G* = Φ_α_, for some α>2 or *G* =Ψ_α_, for α>0. Assume further that {*X_i_*} satisfies the condition

$$\underset{k\to \propto }{\lim}\;k\underset{n\to \propto }{\lim}\sup\;D\prime \left(a_{n},u_{n}\right)=0,$$

where

$$\begin{array}{c} D\prime \left(a_{n},u_{n}\right)=\sum_{j=1}^{n/k}=E[|\exp\left(ita_{n}^{-1}\sum_{\underset{l\neq j}{l=1}}X_{t}\right)\\ -1|\left[\chi \left(X_{l}>u_{n}\right)\right] \end{array}$$ (2)

Then ${\bar{S}}_{n}$ and ${\bar{M}}_{n}$ are asymptotically independent. Local dependence condition *D′*(*a_n_*, *u_n_*) is quite weak and satisfied, for example, by m-dependent sequences and by Gaussian sequences with summable covariances.

### Case 2: Heavy Tailed Case

Assume that

$$\begin{array}{l} 1-F\left(x\right)=px^{-\alpha }\;L\left(x\right),\\ F\left(-x\right)=qx^{-\alpha }\;L\left(x\right) \end{array}$$

where *L*(*x*) is a slowly varying function and 0<α<2. Then for the associated iid sequence (Chow and Teugels [5])

$$\left({\bar{S}}_{n},{\bar{M}}_{n}\right)\to^{d}\left(U,V\right),$$

where

$$E\left(e^{it\upsilon }\chi \left(V\leq \upsilon \right)\right)=W_{\alpha }\left(t,p\right)\exp\int_{\upsilon }^{\infty }e^{itkw}dw^{-\alpha }.$$

Here *χ*(*A*) denotes the indicator function of the event *A*, *W*_α_(*t*, *p*) is the characteristic function of a stable law of index *α* and parameter *p*, and *k* is a constant depending on *α* and *p*. Note that for the heavy tailed case, *U* and *V* are dependent. Can the type of local dependence of the *X*-sequence make a difference to the Chow-Teugels limit? It can! If large values are cancelled by large negative values, then sums and maxima can be asymptotically independent. We show this by constructing an example:

Let {*Y_i_*} be a stationary sequence with

$$1-F_{Y}\left(y\right)=1-y^{-4},$$

ϵ<1, *γ*≥1 and for *ν*>0 such that ϵ + *ν*< 1, let

$$X_{i}=\{\begin{array}{cc} Y_{i} & \text{with probability}\;Y_{i-t}^{-v}\\ -Y_{i-1} & \;\text{with probability}\;1-Y_{i-1}^{-v} \end{array}$$

Then it can be shown that {*X_i_*} is stationary, 1-dependent and 1−*Fx*(x) = ϵ(ϵ + *ν*)^−1^*x*^−ϵ^, *Fx*(−x) = x^−ϵ^. Hence the limit distribution of $\left({\bar{S}}_{n},{\bar{M}}_{n}\right)$ for the associated iid sequence is the Chow-Teugels limit with α=ϵ and *p* = ϵ(2ϵ+*ν*)^−1^. The components of this limit are dependent. However, $\left({\bar{S}}_{n},{\bar{M}}_{n}\right)$ of the dependent {*X_i_*} process can be shown to be asymptotically independent due the cancellation of large positive values by large negative values values, thus showing that local dependence may make a difference on the limit distribution. However, if we rule out this type of cancellation, then the limit distribution is not affected by the dependence in {*X_i_*}, as the following theorem demonstrates (Anderson and Turkman [4]). One possible local condition which rules out this type of cancellation is Davis’ [6] D′(*a_n_*) condition, which we assume in the theorem. This restrictive technical condition also rules out clustering of large and small values above and below certain thresholds. Types of processes which satisfy this condition (and others which we need in the theorem) can be seen in Davis [6].

Let {*E_i_*} be an iid unit mean exponential sequence, $\Gamma_{j}=\sum_{i=1}^{j}E_{i}$ and {δ*_i_*} iid taking values +1 and −1 with probabilities *p* and *q* respectively. Then

**Theorem 2.2**

- If 0<α<1, *p*>0 and conditions *D*(*a_n_*), *D*′(*a_n_*) of Davis [6] hold then
  $$\left({\bar{S}}_{n},{\bar{M}}_{n}\right)\to^{d}\left(\sum_{j=1}^{\propto }\delta_{j}\Gamma_{j}^{-1/\alpha },\Gamma_{D}^{-1/\alpha }\right),$$
  where *D* = min {*j* : δ*_j_* = 1}.
- If 1≤α <2, *p*>0 and conditions *D*(*a_n_*), *D*′ (*a_n_*) and *D^n^*(*a_n_*) of Davis [6] hold then
  $$\begin{array}{l} \left({\tilde{S}}_{n},{\bar{M}}_{n}\right)\to^{d}\\ \left(\sum_{j=1}^{\infty }\left\{\delta_{j}\Gamma_{j}^{-1/\alpha }-\left(p-q\right)E\left(\Gamma_{D}^{-1/\alpha \left(0,1\right)}\right)\right\},\Gamma_{D}^{-1/\alpha }\right). \end{array}$$
- Under the conditions of (i) and (ii),
  $$E\left[e^{it{\bar{S}}_{a}}\chi \left({\bar{M}}_{n}\leq x\right)\right]\to^{d}W_{\alpha }\left(t,p\right)\Phi_{\alpha }\left(x\right)e^{-h\left(t,x\right)},$$
  where
  $$h\left(t,x\right)=\int_{x}^{\infty }\left[e^{it\left(C\delta \right)1/\alpha_{-y}}-1\right]d\left(-y^{-n}\right),$$
  and
  $$\delta =p\frac{2-\alpha }{\alpha },$$
  and *C* is a positive constant. Note that the value of the limit does not depend on the dependence structure of {*X_i_*}.

These results seem to be discouraging for statistical applications. For example, for sequences with finite variance, the independence of (*U*, *V*) does not offer a basis for the use of average wind speeds in inferences about gusts, contrary to the evidence shown in data. This may be due to:

- Time intervals are not long enough in practice for asymptotic results to give adequate approximations,
- The correlation structure of the data is not well represented by our mixing and local dependence conditions,
- Residual seasonality remains in the data. Based on these possible deviations, statistical models establishing connection between means and extreme events are suggested in Anderson and Turkman [3].

## 3. Extremes of Averages Over Different Time Scales

The specific problem to be adressed in this section is as follows: Let *X*, represent the instantaneous value of the environmental variable at time *t*, and denote by *X_T,t_* a moving average of {*X_t_*} over the range *T*:

$$X_{T,t}=\frac{1}{T}\sum_{i=0}^{T-1}X_{t-i},$$ (3)

Then we are interested in the paired series {*X_T,t_*, *X_S,t_*} for different fixed *S* and *T* and in particular, in the joint distributional properties of extremes of the pair {*X_T,t_*, *X_S,t_*}.

We will give results only for sequences with the heavy tailed distributions. The light tailed case is more complicated, since in this case large values of the moving averages may occur due to the contribution of several relatively large values of the sequence in contrast to the heavy tailed case when large values of the moving averages are dominated by the largest value of the sequence. The techniques to be used to study these questions will be developments of those used by Davis and Resnick [8].

Suppose that ${\left\{X_{i}\right\}}_{i=1}^{\infty }$ are iid random variables with

$$P\left(X_{i}>x\right)∼x^{-\alpha }L\left(x\right),$$

where *L*(*x*) is slowly varying as *x*→∞, that is *X* ∈ *D* (Φ_α_). Take constants *a_n_* such that

$$nP\left(X_{i}>a_{n}x\right)\to x^{-\alpha },$$

Consider a point process *P_n_* which puts points at $\left(\frac{k}{n},a_{n}^{-1}X_{k}\right),k=1,2,\ldots$. Hence *P_n_* is the random point measure on sets in *R*^+^ × *R*

$$\sum_{k=1}^{\infty }\in_{\left(\frac{k}{n},a_{n}^{-1}X_{k}\right)}\left(\cdot \right),$$

where

$$\in_{x}\left(A\right)=\{\begin{array}{ll} 1 & \text{if}\;x\in A\\ 0 & \text{if not}. \end{array}$$

It is known that *P_n_* converges weakly as *n*→∞ to a point process *P*, a Poisson process with mean measure *μ*. on *R* ^+^ × (*R*^+^ −{0}). (Davis and Resnick call this Poisson measure, the Poisson random measure *PRM* (µ) and consider a more general case which involve the left tail of the distribution as much as the right tail. Here due to the special simple form of the moving averages, we restrict ourselves to the space (0, ∞).)

Here

$$d\mu =dt\times \alpha x^{-u-1}\in_{x}\left(0,\infty \right)dx.$$

Hence

$$\sum_{k=1}^{\propto }\in_{\left(\frac{k}{n},a_{n}^{-1}X_{k}\right)}\left(\cdot \right)\to \sum_{k=1}^{\infty }\in_{\left(l_{k},j_{k}\right)}\left(\cdot \right),$$

where $\left\{\left(t_{k},j_{k}\right)\right\}\underset{k=1}{\infty }$ are the Points of *P*.

Davis and Resnick [8] show that, correspondingly, for {*X_T,t_*} with same normalization

$$\sum_{k=1}^{\propto }\in_{\left(\frac{k}{n},a_{n}^{-1}X_{T,k}\right)}\left(\cdot \right)\to \sum_{k=1}^{\infty }\sum_{l=1}^{T}\in_{\left(l_{k},j_{k}c_{i}\right)}\left(\cdot \right)$$

on *R* ^+^ ×(*R*^+^−{0}), where

$$c_{i}\{\begin{array}{ll} \frac{1}{T} & i=1,2,\ldots ,T.\\ 0 & \text{otherwise} \end{array}$$

and (t*_k_*, *j_k_*) are as above.

Results for the point process generated simultaneously by {*X_T,t_*} and {*X_s,i_*} processes can be obtained with a straightforward generalization:

$$\sum_{k=1}^{\propto }\in_{\left(\frac{k}{n},a_{n}^{-1}X_{T,k}a_{n}^{-1}\chi_{s,i}\right)}\left(\cdot \right)\to \sum_{k=1}^{x}\sum_{i=1}^{T}\in_{\left(l_{k},j_{k}c_{i},j_{k}c_{i}\right)}\left(\cdot \right)$$ (4)

on *R*^+^×(*R*^+^−{0})^2^, where

$${\bar{c}}_{i}=\{\begin{array}{ll} \frac{1}{S} & i=1,2,\ldots ,S.\\ 0 & \text{otherwise} \end{array}$$

The joint limit distribution of *M_T,n_* and *M_S,n_* now can easily be calculated since

$$\begin{array}{l} P(M_{T,n}\leq a_{n}x,M_{S,n}\leq a_{n}y)\\ =P(\overset{\infty }{\underset{k=1}{\sum }}\in_{\left(\frac{1}{n},a_{n}^{-1}X_{T,k},a_{n}^{-1}X_{s,1}\right)}((0,1]\times ((-\infty ,x]\times (-\infty ,y]{)}^{c})=0)\\ \to P(\overset{\infty }{\underset{k=1}{\sum }}\overset{T}{\underset{i=1}{\sum }}\in_{\left(l_{k},j_{k}c_{i},j_{k}c_{i}\right)}((0,1]\times ((-\infty ,x]\times (-\infty ,y]{)}^{c})=0)\\ =P(\overset{\infty }{\underset{k=1}{\sum }}\in_{\left(t_{k},T^{-1}/k,S^{-1}jk\right)}((0,1]\times ((-\infty ,x]\times (-\infty ,y]{)}^{c})=0)\\ =P(\overset{\infty }{\underset{j=1}{\sum }}\in_{\left(t_{k}j_{k}\right)}((0,1]\times (Tx\Lambda Ts,\infty ))=0)\\ =\exp[-\mu ((0,1]\times (Tx\Lambda Sy,\infty ))]\\ =\exp[-\overset{\infty }{\underset{Tx\wedge Sy}{\int }}q(x)dx]\\ =\{\begin{array}{ll} \exp[-p{\left(Tx\Lambda Sy\right)}^{-\alpha }] & \text{if}\;Tx\Lambda Sy>0\\ 0 & \text{if not} \end{array} \end{array}$$ (5)

### Special Cases

[1] *S* = *T*. Then

$$P\left(a_{n}^{-1}M_{T,n}\leq x,a_{n}^{-1}M_{S,n}\leq y\right)=P\left(a_{n}^{-1}M_{T,n}\leq x\Lambda y\right),$$

so from theorem 3.1 of Davis and Resnick [8], we should have

$$P\left(a_{n}^{-1}M_{T,n}\leq x,a_{n}^{-1}M_{S,n}\leq y\right)=\to e^{-p{\left(T\left(x\Lambda y\right)\right)}^{-\alpha }}.$$

Hence the result Eq. (5) is consistent with the existing results on this special case.

[2] *S* = 1 <*T*, so M*_S,n_* = max*_i_*_≤_*_n_ X_i_*, and the result Eq. (5) says that

$$P\left(a_{n}^{-1}M_{T,n}\leq x,a_{n}^{-1}M_{S,n}\leq y\right)\to e^{-p{\left(Tx\Lambda y\right)}^{-\alpha }}.$$

Note that the above distribution is the joint limit distribution of the maximum of the {*X_i_*} process and the maximum of a moving average of it.

The above set up is a very simple case. The immediate question is: what if the {*X_i_*} are themselves a dependent sequence?

We can get a partial answer by taking {*X_i_*} to be itself a moving average:

$$X_{i}=\sum_{j=1}^{\infty }a_{j}Z_{i-j},$$

say, where *Z_i_* satisfy the same conditions as the *X_i_* and *a_j_*≥0 (for simplicity)Thus

$$X_{T,i}=\left\{X\right\}*\left\{\frac{1}{T}\right\},$$

and

X={Z}*{a}.

Hence

$$X_{T,i}=\left\{Z\right\}*\left\{a\right\}*\left\{\frac{1}{T}\right\}=\left\{Z\right\}*\left\{d\right\},$$

say, where

$$d=\left\{a\right\}*\left\{\frac{1}{T}\right\}.$$

Note that for any *l* = 1,2,…,

$$d_{l}=\frac{1}{T}\sum_{i=1}^{T\Lambda l-1}a_{l-i},i=1,2,\ldots$$

As before

$$\begin{array}{l} P\left(a_{n}^{-1}M_{T,n}\leq x,a_{n}^{-1}M_{S,n}\leq y\right)\\ \to P(\sum_{k=1}^{\propto }\sum_{i=1}^{\propto }\in_{\left(i_{k},f_{k}d_{0}j_{k}{\bar{d}}_{i}\right)}(\left(0,1\right]\times \\ (\left(-\infty ,x\right]\times {\left(-\infty ,y\right])}^{c})=0), \end{array}$$ (6)

where

$${\bar{d}}_{i}=\frac{1}{S}\sum_{i=1}^{S\Lambda I-1}a_{l-i}.$$

Note that

$$\begin{array}{c} \left\{\sum_{i=1}^{\propto }\in_{\left(l_{k},j_{k}{\bar{d}}_{i}j_{k}d_{1}\right)}\left(\left(0,1\right]\times {\left(\left(-\infty ,x\right]\times \left(-\infty ,y\right]\right)}^{c}\right)=0\right\}\equiv \\ \left\{j_{k}\left({\bar{d}}_{i},{\bar{d}}_{i}\right)\in \left(-\infty ,x\right]\times \left(-\infty ,y\right]\right\}, \end{array}$$

for every *i* for which one of the components is non-zero. This is the case iff *j_k_d*^+^≤*x* and *j_k_d*^+^≤*y*, where *d*^+^ = max *d_ì_* and *d*^+^ = max *d_i_*. Hence the limit of Eq. (6) is given by

$$\begin{array}{l} P\left(\sum_{k=1}^{\infty }\in j_{k}\left(\frac{x}{d^{+}}\Lambda \frac{y}{d^{+}},\infty \right)=0\right)\\ =e^{-\int }\frac{x}{d+}\Lambda {\frac{y}{d+}}^{q\left(x\right)dx}\\ \begin{array}{ll} =\{\begin{array}{l} e^{-p{\left(\frac{x}{d_{+}}\Lambda \frac{y}{d_{+}}\right)}^{-e}}\\ 0 \end{array} & \begin{array}{l} \text{if}\left(\frac{x}{d^{+}}\Lambda \frac{y}{d^{+}}\right)>0\\ \text{otherwise} \end{array} \end{array} \end{array}$$ (7)

Hence we see that this kind of dependence in the underlying process does not change the form of the limit distribution. It would be interesting to obtain similar results for general stationary sequences. This could be done by using characterization of the limit point processes for the sequence of point processes with points $\left(t/n,a_{n}^{-1}X_{i}t=1,2,\ldots ,n\right)$ given by Davis and Hsing [7] when {*X_t_*} is a stationary sequence with regularly varying tails. In their paper, Davis and Hsing show that when {*X_i_*} satisfies a proper mixing condition then

$$N_{n}=\sum_{t=1}^{n}\in_{\left(\frac{l}{n},a_{n}^{-1}X_{i})\right)}\left(\cdot \right)$$

converges to a point process *N* of the form

$$N=\sum_{i=1}^{\infty }\sum_{j=1}^{\infty }\in_{\left(S_{n}P_{t}Q_{ij}\right)},$$

where

$$\sum_{t=1}^{\infty }\sum_{j=1}^{\propto }\in_{\left(S_{t},P_{t}\right)}$$

is PRM(*ν*) with

$$dv=dt\times \gamma \alpha x^{-\alpha -1}dx,x>0,$$

(This is slightly weaker form of Davis-Hsing limit, since we consider the convergence only on R^+^ −{0} not involving the left tail.) They also show that under the proper mixing condition, the convergence of *N_n_* to *N* is equivalent to:

For *r_n_* → ∞, *r_n_/n* →0 as *n* → ∞, and *k_n_* = [*n/r_n_*],

$$\underset{n\to \infty }{\lim}k_{n}\;P\left(\underset{1\leq i\leq r_{n}}{\max}X_{j}>a_{n}x\right)=\gamma x^{-\alpha },$$

and

$$\begin{array}{l} P\left(\sum_{j=1}^{r_{n}}\in_{X_{j}/\max_{1\leq i\leq r_{n}}X_{J}}\in ./\underset{1\leq i\leq t_{n}}{\max}X_{j}>a_{n}x\right)\\ \to Q\left(.\right) \end{array}$$

Here *Q*(.) is the distribution of the iid point processes

$$\left\{\sum_{j=1}^{\propto }\in_{Qtj}\left(\cdot \right)\right\}\underset{t=1.}{\infty }$$

From this basic result it may be possible to obtain the limiting form of

$$N_{n}^{t}=\sum_{t=1}^{n}\in_{\left(\frac{t}{n},a_{n}^{-1}X_{T,t},a_{n}^{-1}X_{S,t}\right)}\left(\cdot \right),$$

where

$$X_{T,t}=\frac{1}{T}\sum_{j=1}^{T}X_{t-j},$$

and

$$X_{S,t}=\frac{1}{S}\sum_{j=1}^{S}X_{t-j}.$$

One would expect that if the above sequence of point processes converges, then the limit point process should be of the form

$$\begin{array}{l} N_{n}^{\prime }\to \\ \overset{\infty }{\underset{t=1}{\sum }}\overset{\infty }{\underset{j=1}{\sum }}\overset{\infty }{\underset{i=1}{\sum }}\in_{(S_{t},c_{i},P_{i}Q_{ij},c_{i}^{t}P_{i}Q_{tj}),} \end{array}$$

where

$$\begin{array}{l} c_{i}=\{\begin{array}{ll} \frac{1}{T} & i=1,2\ldots ,T\\ 0 & \text{otherwise} \end{array}\\ {c^{\prime }}_{i}=\{\begin{array}{ll} \frac{1}{S} & i=1,2\ldots ,S\\ 0 & \text{otherwise} \end{array} \end{array}$$

Straightforward adjustment of Resnick’s [11] arguments which involve consecutive application of the continuous mapping theorem is not possible, since these arguments use the convergence of

$$nP\left(a_{n}^{-1}X_{1}>\delta ,a_{n}^{-1}X_{2}>\delta \right)\to 0,\delta >0$$

which is clearly not satisfied by most sequences with strong local dependence.
